# Postoperative pain control in orthopedic surgery: A randomized trial comparing regional anaesthesia and systemic opioids

**DOI:** 10.6026/9732063002001931

**Published:** 2024-12-31

**Authors:** J. Naveen Bose, Kaushik Rajavel, Rakshana Munusamy, Priyadarshini Ramesh

**Affiliations:** 1 Department of Emergency Medicine, Madurai Medical College, Madurai, Tamil Nadu, India; 2 Department of Orthopaedics, Madras Medical College, Chennai, Tamil Nadu, India; 3 Department of Surgery, Madurai Medical College, Madurai, Tamil Nadu, India; 4 Department of Obstetrics & Gynaecology, Madras Medical College, Chennai, Tamil Nadu, India

**Keywords:** Postoperative pain, orthopedic surgery, regional anaesthesia, systemic opioids, pain control, opioid consumption, functional recovery

## Abstract

Effective pain management is crucial for recovery after orthopedic surgery, influencing outcomes, hospital stay duration and patient satisfaction. This randomized trial compared regional anesthesia (RA) with systemic opioid therapy for postoperative pain control in 100 patients undergoing major orthopedic surgery. RA resulted in significantly lower pain scores at 12, 24 and 48 hours post-surgery (p < 0.001) and reduced opioid consumption (p < 0.001). Adverse effects like nausea and vomiting were more common in the opioid group (p = 0.002), while functional recovery was superior in the RA group (p = 0.015). The length of hospital stay was similar between groups (p = 0.116). These findings indicate that RA is more effective than systemic opioids for postoperative pain management, with fewer side effects and improved functional recovery, making it a preferred option in orthopedic surgery.

## Background:

Pain control in the postoperative period is an important part of recovery after major orthopaedic interventions, such as joint arthroplasty or spinal surgery [1]. Good pain management not only provides relief but functions as a significant aid for early mobilization, shortening the period of stay and preventing complications such as deep vein thrombosis and pulmonary embolism from occurring post-surgery [2]. This stage of pain management directly correlates with short-term and long-term outcomes such as patient satisfaction, rehabilitation progress and functional recovery. Poor pain control is also associated with chronic pain syndromes, delayed recovery and dependency on long-term medications [3, 4]. Traditionally, systemic opioids have been used for the treatment of moderate to severe postoperative pain. Central nervous system action of drugs, especially morphine, hydromorphone and oxycodone, serves the purpose of pain relief and is effective for the treatment of severe pain in many kinds of surgical procedures [5]. However, widespread use of these drugs has opened many serious concerns due to widely reported side effects of the drugs. These include respiratory depression, which is life-threatening; nausea and vomiting; constipation; pruritus; and urinary retention-all of which can compromise recovery and comfort in these patients [6]. Yet, far more ominous is the potential for opioid dependency and the attendant increased morbidity and mortality with opioids, which now constitutes a critical public health crisis around the globe to underscore the need for alternatives in postoperative pain management [7]. Such concerns have made RA techniques tops in applications that have gained the majority in systemic opioids. The RA technique is also a technique that prevents the conduction of nervous impulses within given areas of the body in the blockade of nerves to not allow the transmission of pain stimuli from the surgical site towards the CNS [8]. This is through creating minimal involvement of systems, which enables effective pain relief without most side effects as usually associated with opioids. Some of the techniques used in orthopaedic surgery are peripheral nerve blocks, spinal anesthesia and epidural anesthesia. These methods have been associated with reduced opioid consumption, low incidence of opioid-related side effects and improved patient satisfaction [9]. The advantages of RA compared to systemic opioids go beyond the effective control of pain. Besides reducing opioid intake, RA can possibly reduce the likelihood of opioid dependency, which has become a related and rising concern in the management of postoperative pain [10]. It was reported that better pain relief by RA was associated with earlier mobilization, faster rehabilitation and better functional outcomes in orthopedic patients. Currently, there are only a few high-quality randomized controlled trials, which have made recommendations on what would constitute an effective comparison of RA efficacy compared to systemic opioids in this context [11]. This study will fill such gaps by comparing postoperative pain management from major orthopedic surgeries under regional anesthesia compared with systemic opioids. It will focus on key outcomes: relief of pain, rate of opioid consumption-including the rate of opioid-related side effects-and impact on functional recovery. Therefore, it is of interest to document optimal pain management in orthopedic surgery during or shortly after the time of its intervention, with a view to guiding clinical practice toward better and safer approaches.

## Methodology:

This is a randomized controlled trial conducted between January 2023 and December 2023 with 100 patients with major orthopedic surgeries such as total knee replacement, hip replacement and spinal surgery.

## Inclusion criteria:

[1] Patients aged 18 to 75 years undergoing elective orthopedic surgery.

[2] No contraindications to regional anesthesia or opioid use.

## Exclusion criteria:

[1] Patients with chronic pain conditions or opioid use disorders.

[2] Patients with contraindications to anesthesia or opioid use.

## Study design:

## Patients were randomly assigned to:

[1] Group A (Regional Anesthesia): Received regional anesthesia (spinal, epidural, or peripheral nerve blocks).

[2] Group B (Systemic Opioids): Received systemic opioid therapy (morphine or oxycodone via patient-controlled analgesia).

## Data collection:

[1] Pain Intensity: Assessed using the Visual Analog Scale (VAS) at 12, 24 and 48 hours after surgery.

[2] Opioid Intake: Measured in morphine milligram equivalents for 48 hours.

[3] Side Effects: Opioid-related side effects frequency, such as nausea, vomiting, constipation and respiratory depression.

[4] Functional Recovery: Recovery to early mobilization and recovery to basic activity by the postoperative functional score.

[5] Hospital Stay: Measured in days.

[6] Statistical Analysis Data were analysed using SPSS software and continuous variables were reported as mean ± SD while the categorical variables were presented as percentages. A p < 0.05 was considered statistically significant.

## Results:

This randomized controlled trial was conducted between January 2023 and December 2023 with 100 patients who were subjected to major orthopedic surgeries consisting of total knee replacement, hip replacement and spinal surgery. Both groups were homogenous on all characteristics at baseline; thus, demographic factors played no role in the result (Table 1). All the points of follow-up were the case for RA as compared with the opioid group for pain scores (Table 2). The number of consumptions of opioid in the regional anesthesia group was very significantly less than that of the systemic opioid group (Table 3). The opioid group showed significantly more side effects, especially nausea, vomiting and constipation (Table 4). The patients in the regional anesthesia group had better early functional recovery at 24 and 48 hours following surgery compared to the opioid group (Table 5). The patients who received regional anesthesia were mobilized earlier than the systemic opioid group and it aids in faster recovery (Table 6).

Postoperative complication rates were equivalent between groups, however urinary retention more commonly occurred in the opioid group (Table 7). Higher patient satisfaction with pain management was reported in the regional anesthesia group compared to the systemic opioids group (Table 8). The duration of hospital stay did not have a statistical difference between the two groups (Table 9). Greater opioid-related side effects were observed in the systemic opioid group when compared to the regional anesthesia group (Table 10).

## Discussion:

This study proves that regional anesthesia is better than systemic opioids after postoperative pain after major orthopedic surgery [12]. Generally, patients who received regional anesthesia had scores for very significantly lower postoperative pain and fewer narcotics compared to patients in the systemic opioid group. A smaller rate of opioid side effects in the RA group, which are nausea, vomiting and constipation, puts this in prominence as serious complications may be triggered by opioids [13]. This study, besides the effect on pain relief, has made better functional recovery and earlier mobilization. Undoubtedly, these are the benefits that regional anesthesia brings about, most importantly. Early mobilization is especially important in orthopedic surgery because it prevents complications such as deep vein thrombosis and accelerates rehabilitation [14]. Where-as No difference is noted in length of stay within groups, patients who received regional anesthesia are seen to have more satisfaction rates concerning pain management most probably due to reduced side effects and effective pain control [15]. However, several factors significantly decide success, such as the skills of anaesthesiologists, the medical conditions of patients and the contraindications of patients. For instance, some patients may have contraindications for regional anesthesia; therefore, regional anesthesia is only applicable to a few populations [16]. This study is consistent with studies that exist in agreement with that regional anesthesia lowers the ingestion of opioids, facilitates management of postoperative pain and limits adverse impacts related to pain during the early periods of the postoperative stage [17]. There is more research to be done with the aim of clearly defining the long-term benefits of RA postoperative surgeries on patients and populations and the utilization for reducing opioid dependency following a surgery. A systematic review identified 21 studies comparing paracetamol alone or in combination with other NSAIDS and reported increased efficacy with the combination of two agents than with either alone [18].

## Conclusion:

This randomized trial clearly showed that regional anesthesia provides better postoperative pain relief compared with systemic opioids in orthopedic surgery; indeed, it reduces opioid intake, reduces side effects and accelerates functional recovery. There were no differences in the hospital stays between the two techniques, but the patients operated under regional anesthesia exhibited greater satisfaction with pain control. The conclusion is that regional anesthesia is the method of choice for postoperative pain control in major orthopedic surgeries.

## Figures and Tables

**Table 1:** The baseline characteristics of patients

**Characteristic**	**Group A (Regional Anesthesia)**	**Group B (Systemic Opioids)**	**p-value**
Age (Mean ± SD)	62.5 ± 7.8	63.1 ± 8.1	0.584
Gender (Male)	29:21:00	30:20:00	0.812
Type of Surgery	Hip (48%), Knee (40%),	Hip (50%), Knee (38%),	0.932
	Spine (12%)	Spine (12%)	

**Table 2:** Postoperative pain scores (VAS)

**Time Post-Surgery**	**Group A (Regional Anesthesia)**	**Group B (Systemic Opioids)**	**p-value**
12 Hours	2.9 ± 0.8	5.3 ± 1.1	<0.001
24 Hours	2.5 ± 0.9	4.9 ± 1.2	<0.001
48 Hours	2.1 ± 0.7	4.4 ± 1.1	<0.001

**Table 3:** Levels of opioid consumption (Morphine milligram equivalents)

**Group**	**Total Opioid Consumption (Mean ± SD)**	**p-value**
Group A (Regional)	17 ± 6 mg	
Group B (Opioids)	44 ± 9 mg	<0.001

**Table 4:** The incidence of adverse effects

**Adverse Effect**	**Group A (Regional Anesthesia)**	**Group B (Systemic Opioids)**	**p-value**
Nausea and Vomiting	10%	32%	0.002
Constipation	8%	22%	0.008
Respiratory Depression	0%	6%	0.032

**Table 5:** Functional recovery scores (1-10 Scale)

**Group**	**Functional Score at 24 Hours**	**Functional Score at 48 Hours**	**p-value**
Group A (Regional)	6.2 ± 1.4	7.8 ± 1.2	
Group B (Opioids)	4.5 ± 1.5	6.0 ± 1.3	<0.001

**Table 6:** The time to first mobilization (Hours)

**Group**	**Time to Mobilization (Mean ± SD)**	**p-value**
Group A (Regional)	10.2 ± 2.4	
Group B (Opioids)	13.4 ± 3.1	0.003

**Table 7:** Postoperative complications

**Complication Type**	**Group A (Regional Anesthesia)**	**Group B (Systemic Opioids)**	**p-value**
Urinary Retention	4%	12%	0.056
Infection	6%	8%	0.455
Deep Vein hrombosis	2%	4%	0.612

**Table 8:** Highlights patient satisfaction scores (1-5 Scale)

**Group**	**Satisfaction Score (Mean ± SD)**	**p-value**
Group A (Regional)	4.7 ± 0.5	
Group B (Opioids)	3.8 ± 0.7	<0.001

**Table 9:** The length of hospital stays (Days)

**Group**	**Mean Length of Stay (Mean ± SD)**	**p-value**
Group A (Regional)	4.1 ± 1.3	
Group B (Opioids)	4.4 ± 1.6	0.116

**Table 10:** Total opioid-related side effects (Composite Measure)

**Group**	**Number of Side Effects Per Patient (Mean ± SD)**	**p-value**
Group A (Regional)	0.8 ± 0.4	
Group B (Opioids)	2.1 ± 1.0	<0.001
